# Impaired Hyperemic Response to Exercise Post Stroke

**DOI:** 10.1371/journal.pone.0144023

**Published:** 2015-12-02

**Authors:** Matthew J. Durand, Spencer A. Murphy, Kathleen K. Schaefer, Sandra K. Hunter, Brian D. Schmit, David D. Gutterman, Allison S. Hyngstrom

**Affiliations:** 1 Department of Physical Medicine and Rehabilitation, Medical College of Wisconsin, Milwaukee, Wisconsin, 53226, United States of America; 2 Department of Medicine–Cardiovascular Center, Medical College of Wisconsin, Milwaukee, Wisconsin, 53226, United States of America; 3 Department of Biomedical Engineering, Marquette University, Milwaukee, Wisconsin, 53201, United States of America; 4 Department of Physical Therapy, Marquette University, Milwaukee, Wisconsin, 53201, United States of America; Emory University School Of Medicine, UNITED STATES

## Abstract

Individuals with chronic stroke have reduced perfusion of the paretic lower limb at rest; however, the hyperemic response to graded muscle contractions in this patient population has not been examined. This study quantified blood flow to the paretic and non-paretic lower limbs of subjects with chronic stroke after submaximal contractions of the knee extensor muscles and correlated those measures with limb function and activity. Ten subjects with chronic stroke and ten controls had blood flow through the superficial femoral artery quantified with ultrasonography before and immediately after 10 second contractions of the knee extensor muscles at 20, 40, 60, and 80% of the maximal voluntary contraction (MVC) of the test limb. Blood flow to the paretic and non-paretic limb of stroke subjects was significantly reduced at all load levels compared to control subjects even after normalization to lean muscle mass. Of variables measured, increased blood flow after an 80% MVC was the single best predictor of paretic limb strength, the symmetry of strength between the paretic and non-paretic limbs, coordination of the paretic limb, and physical activity. The impaired hemodynamic response to high intensity contractions was a better predictor of lower limb function than resting perfusion measures. Stroke-dependent weakness and atrophy of the paretic limb do not explain the reduced hyperemic response to muscle contraction alone as the response is similarly reduced in the non-paretic limb when compared to controls. These data may suggest a role for perfusion therapies to optimize rehabilitation post stroke.

## Introduction

Following stroke, blood flow to the musculature of the paretic limb is decreased at rest compared to the non-paretic limb and the limbs of healthy subjects.[1–3] Presumably, the reduction in paretic limb blood flow is due to both muscle atrophy caused by reduced neural drive to the affected limb as well as deconditioning due to decreased use of the paretic limb. [4, 5] Metabolic changes such as augmented lactate production and reduced oxygen uptake have also been reported in the paretic muscle during low level exercise.[6] These observations, coupled with deficient central neural activation of the paretic muscle during exercise,[7] combine to severely limit paretic lower limb function in this subject population and limit the potential impact of rehabilitation on motor recovery.

To date, the hyperemic response to paretic limb muscle contraction has not been examined in the chronic stroke population. The purpose of this study was to examine femoral artery blood flow in both the paretic and non-paretic lower limb of stroke survivors, and in neurologically intact control subjects, in response to graded, submaximal contractions of the knee extensor muscles. In healthy subjects peripheral blood flow is tightly matched to the metabolic demand of exercising muscle. Given the reduced neural activation of the paretic musculature [7, 8] and a shift in paretic muscle physiology to favor a more fatigue-prone state post stroke,[6, 9] we hypothesize that the hyperemic response to contractions of the knee extensor muscles will be blunted in the paretic limb of subjects with chronic stroke, and that subjects with a more robust hyperemic response will have greater lower limb function and strength.

## Materials and Methods

### Subjects

All activities in this study were approved by the Institutional Review Boards of Marquette University and the Medical College of Wisconsin. All participants gave written informed consent prior to study participation. Ten participants with chronic stroke (≥ 6 months) and ten age- and sex-matched, neurologically intact subjects were recruited (see Table 1 for details). Stroke subject inclusion criteria: 1) history of a single, unilateral stroke and 2) the ability to ambulate at least 30 feet with or without an assistive device. Stroke subject exclusion criteria: 1) history of multiple strokes, 2) brainstem stroke, 3) any uncontrolled medical condition, 4) lower extremity contractures, 5) resting systolic blood pressure ≥140 mmHg or 6) inability to follow 2–3 step commands. One control subject refused the body composition scan (see below), and non-paretic lower limb blood flow could not be quantified for one stroke subject during the equal torque test session. Subjects were not instructed to abstain from taking their current medications.

**Table 1:** Characteristics of all Subjects.

Characteristic	Control (n = 9)	Stroke (n = 10)
Sex, Male	6	6
Age (yr)	60±6	63±7
Height (cm)	173.2±14.7	172.1±11.7
Weight (kg)	80.3±14.6	85.9±19.7
Body Mass Index (kg/m^2^)	27±4	29±4
Total Body Fat (%)	35.8±6.5	39.8±4.5
Estimated Visceral Fat (%)	27.3±10.5	34.0±11.7
Waist Circumference (cm)	94.4±7.7	103.8±11.4
Hip Circumference (cm)	103.4±6.5	107.8±4.0
Waist to Hip Ratio	0.91±0.04	1.00±0.08
Total Cholesterol (mg/dl)	200±22*	170±32
LDL Cholesterol (mg/dl)	123±26*	91±26
HDL Cholesterol (mg/dl)	60±21	59±21
Triglycerides (mg/dl)	94±50	104±48
Systolic Blood Pressure (mmHg)	125±9	123±15
Diastolic Blood Pressure (mmHg)	80±11	74±9
Heart Rate (bpm)	73±18	75±8
Fugl-Meyer Score	NA	23±7
Physical Activity (Met-h/week)	14±7	13 ±7

All values are expressed as mean ± SD. HDL, high density lipoprotein; LDL, low density lipoprotein; n, number of subjects.

*Significant difference (*p*<0.05) Stroke vs. Control–unpaired t-test.

### Torque Measurements

Participants sat on a Biodex chair with their tested knee and hip flexed to 90°. The isometric torque of the knee extensors was measured with a load cell (JR3 force-torque transducer) sampled at 1000 Hz. Please see below in the Experimental Protocol for detailed description of torque measurements.

### Vascular Measurements

All experimental protocols were performed in a temperature controlled room between 8:00 and 10:00 AM. Subjects were seated in an upright position in the Biodex dynamometer (see below) and rested for a minimum of 15 minutes prior to assessments of blood flow. Subjects had a belt placed around their waist to reduce movement, and all vascular measurements were taken on the inner thigh by the same individual who stabilized the ultrasound probe by hand. The diameter, mean blood flow velocity, maximum blood flow velocity, and calculated volume of blood flow through the superficial femoral artery were measured and analyzed using a Vivid e ultrasound machine (General Electric, Fairfield, CT) equipped with a linear array 4.0–12.0 MHz transducer designed for vascular imaging with an isonation angle of 60°. Five unique measurements consisting of 3 complete cardiac cycles per measurement were averaged prior to testing the MVC of the lower limb (see below) to establish resting values. Immediately following each submaximal contraction subjects remained seated in the upright position in the biodex dynamometer still while a ten second video clip of the artery was recorded. Because subjects were secured to the chair with a lap belt and the isometric contractions do not result in movement of the lower limb, the same portion of the artery was able to be visualized following all contractions. Because local blood flow is tightly coupled to metabolic demand and blood flow rapidly returns to baseline following muscle contractions, only measurements obtained during the first three complete cardiac cycles following the submaximal contractions were included for analysis. To account for atrophy of the paretic limb, blood flow through the femoral artery was normalized to lean muscle mass of the whole lower limb as determined by dual-energy X-ray absorptiometry (DXA) analysis (below). To describe conditions the endothelium of the superficial femoral artery was exposed to following muscle contractions, peak shear stress through the femoral artery was calculated using the equation SS = 8μV_Peak_/D where SS is shear stress, μ is blood viscosity (estimated to be 0.035 dyne x s/cm^2^), V is peak flow velocity, and D is femoral artery diameter.[10, 11]

### Body Composition and Clinical Measurements

All anthropomorphic measurements were performed in triplicate by a licensed bionutritionist. Body composition analysis to determine the estimated visceral fat percentage, total lower limb mass, lean muscle mass of the limbs, and percent fat composition of each limb was conducted using an iDXA (GE Lunar Medical Systems, Madison, Wisconsin). Lower extremity Fugl-Meyer (a quantitative assessment of motor impairment) and ten meter walk tests were performed by a licensed physical therapist. Each subject completed a physical activity questionnaire (estimates Mets-h/week).[9]

### Experimental Protocol

Subjects were seated in a Biodex dynamometer chair with their hips and knees at 90 degrees of flexion. Torso and lap belts were secured around the participant to prevent compensatory movements. The lower limb was securely stabilized by straps into the dynamometer attachment. After resting femoral artery blood flow measurements were made, each participant performed 3–5 isometric knee extension MVCs (5 s each). MVC measurements were made in the right lower limb of controls and both lower limbs of subjects with stroke. The peak knee extension torque from the trials was used as the MVC. Subjects were instructed to kick as “hard and as quickly” as possible. MVC attempts were stopped when subjects repeatedly kicked within 3% of maximal torque recorded. Subjects were given verbal encouragement during each MVC attempt. One minute rests were given between each attempt. Subjects then completed a single 10 second isometric contraction at 20, 40, 60, and 80% of the peak MVC (order randomized). A 10 second contraction was chosen (vs. longer or repeated contractions) in order to examine load-dependent hyperemic responses while minimizing muscle fatigue. Visual and verbal feedback was given throughout the duration of the kick to all subjects. A one minute rest was given between contractions. Blood flow and heart rate were measured immediately prior to each subsequent sub-maximal contraction trial to confirm blood flow values returned to baseline values.

Stroke subjects performed two sets of submaximal contractions in the non-paretic lower limb. During the first set, the target torque matched torque values of the paretic limb at each graded intensity (“equal torque”). This allowed for comparison of flow while each limb performed a similar amount of work despite different amounts of effort. The second set of submaximal contractions were performed based on the measured MVC of the non-paretic lower limb (“equal effort”). This protocol allowed for comparison of flow following similar graded levels of effort, resulting in different degrees of work. To prevent muscle fatigue, the paretic and non-paretic limbs were tested on separate days. The order of testing (non-paretic vs. paretic limb) was counterbalanced.

### Data Processing

Data processing was performed in Matlab (Mathworks, Natick, MA). Torque was zero phased lowpass filtered at 10 Hz using a 2^nd^ order Butterworth filter prior to analysis. MVC amplitude was recorded as the average force during a 100 ms window surrounding the peak torque. An MVC ratio was calculated between the paretic and non-paretic lower limb to assess asymmetry of strength (paretic MVC/non-paretic MVC). The average torque for each 10 second contraction was found by calculating the mean torque value between 2 and 8 seconds of the contraction.

### Statistical Analysis

All data are reported as mean ± SD. A student’s t-test was used to test for differences between control and stroke subjects for the subject characteristics. Separate one-way analysis of variances (ANOVAs) were used to test for differences in lower limb composition and the blood flow response to exercise between the paretic, non-paretic, and control limbs. Differences between individual means after ANOVA were determined using a post hoc Tukey’s test. To test for differences in lower limb composition between the paretic and non-paretic limbs of stroke subjects only, a paired t-test was performed. A mixed model repeated measures ANOVA was used to detect statistical differences in normalized blood flow between the tested limb (non-paretic, paretic and control) and load level (baseline, 20%, 40%, 60%, and 80% MVC). Separate Pearson correlation coefficients were calculated to determine relationships between femoral artery blood flow and the following variables: MVC, MVC ratio between limbs, Fugl-Meyer score, and physical activity. Statistical analyses were performed in SPSS 20.0 (IMB, Armonk, NY). Separate multiple regressions (forward stepwise method) were performed to identify the single best predictor (dependent variables: ml/min/kg lean muscle mass at rest, 20%, 40%, 60%, and 80% MVC) of the following independent variables in the individuals with stroke: Fugl-Meyer score, paretic MVC, MVC ratio, and level of physical activity. Normality of the data from the separate groups was evaluated by visual inspection of Q-Q plots and the Kolmogorov-Smirnov test (normal distribution was accepted with *p* values > 0.05). Separate regression analysis was performed to determine the linear relationship between torque generated and the hyperemic response for the paretic and control limbs (*α* = 0.05). To assess differences in the slope magnitude of these lines, we first examined the respective overlap of the 95% confidence intervals of b_1_ co-efficient calculated for each line. We then used a t-test to determine the probability that the slopes of the 2 lines were different (*α* = 0.05). For all analyses, significance was accepted at *p* < 0.05.

## Results

Subject characteristics are presented in Table 1. Control subjects had higher LDL and total cholesterol than stroke subjects. The average time post-stroke was 14.3±7.1 years. Of the ten stroke subjects, eight had a middle cerebral artery stroke while two had a stroke in the posterior cerebral artery. Eight of the ten subjects were left side affected. Medications that all subjects were taking are listed in S1 Table. Control subjects were not taking any medications.

### Leg Strength, Size, Composition and Resting Blood Flow

Consistent with previous studies performed in stroke subjects,[8] the MVC of the paretic lower limb was lower compared to either the non-paretic lower limb or the lower limbs of age and sex-matched control subjects (Table 2). Further, compared with the non-paretic limb, the paretic limb was (1) significantly smaller, (2) had reduced muscle mass, (3) a higher fat percentage, and (4) reduced femoral artery diameter at rest (Table 2). Absolute blood flow (ml/min) was not significantly different amongst any of the groups at rest (Table 3). In this cohort of subjects, when resting blood flow was normalized to lean muscle mass of the limb, blood flow was 8% lower in the paretic limb compared to the non-paretic limb, however this difference was not statistically significant (*p* = 0.32). Compared to control subjects, the resting blood flow (when normalized to lean muscle mass) was significantly lower in the paretic leg compared to control subjects.

**Table 2:** Leg strength, size and composition of all subjects.

Characteristic	Control (n = 9)	Non Paretic (n = 10)	Paretic (n = 10)
Maximum Voluntary Contraction (Nm)	134.4±48.1*	87.2±53.0	50.6±31.3^#^
Thigh Circumference (cm)	54.7±4.6	53.1±3.9	51.2±4.7
Calf Circumference (cm)	37.5±3.2	36.7±3.5	34.5±2.5
Total Lower Limb Mass (kg)	13.0±2.6	14.3±2.9	13.0±0.8^#^
Lean Muscle Mass of Lower Limb (kg)	8.4±2.7	9.0±2.7	7.8±2.3^#^
Fat Mass of Lower Limb (kg)	4.1±1.3	4.7±0.6	4.7±0.6
Fat Tissue in Lower Limb (%)	32.5±9.9	35.8±8	38.4±7.3^#^
Femoral Artery Diameter (mm)	6.46±1.11	6.03±1.23	5.08±1.04^#^

All values are expressed as mean ± SD. n, number of subjects.

*Significant difference (*p*<0.05) Control vs. Paretic–one way ANOVA.

^#^Significant difference (*p*<0.05) Paretic vs. Non Paretic–paired t-test

**Table 3:** Vascular measurements during submaximal isometric contraction protocol.

Condition	Test Limb	Percent Maximum Voluntary Contraction				
		Rest	20	40	60	80
Heart Rate (bpm)	Control	73±14	76±13	79±14	80±15	81±18
	Non Paretic—Equal Effort	73±8	73±11	77±8	75±9	80±11
	Non Paretic—Equal Torque	NA	75±12	74±7.5	76±8	77±10
	Paretic	73±8	73±10	74±8	78±10	77±10
Femoral Artery Diameter (mm)	Control	6.46±1.11	6.49±1.09	6.47±1.11	6.47±1.09	6.48±1.11
	Non Paretic—Equal Effort	6.03±1.23	6.07±1.25	6.00±1.24	6.04±1.27	6.06±1.22
	Non Paretic—Equal Torque	NA	6.10±1.15	6.08±1.24	6.11±1.23	6.10±1.18
	Paretic	5.08±1.08*	5.09±1.12*	5.10±1.21	5.05±1.11*	5.04±1.12*
Mean Blood Flow Velocity (cm/s)	Control	6.9±2.0	11.3±4.2	13.1±5.0	15.2±6.6	19.0±4.4
	Non Paretic—Equal Effort	6.2±2.5	8.1±3.1	11.0±4.7	12.7±1.9	13.7±5.0
	Non Paretic—Equal Torque	NA	7.2±3.2*	9.6±4.0	12.2±7.3	11.8±3.7
	Paretic	6.1±1.7	7.2±1.8*	9.9±3.5	10.6±3.6	11.0±3.8*
Peak Blood Flow Velocity (cm/s)	Control	62.5±11.8	69.6±13.8	74.1±17.1	83.2±11.7	87.2±17.9
	Non Paretic—Equal Effort	65.2±21.7	67.3±20.6	68.9±20.4	72.4±22.8	77.9±5.9
	Non Paretic—Equal Torque	NA	67.3±3.6	69.0±7.0	75.5±8.6	72.7±14.5
	Paretic	62.2±17.0	64.9±11.1	73.0±13.9	70.9±11.5	74.4±15.2
Blood Flow (mL/min)	Control	136±56	260±99	343±137	388±89	443±172
	Non Paretic—Equal Effort	110±69	161±92	207±110	237±118*	264±135*
	Non Paretic—Equal Torque	NA	129±51*	171±73*	205±112*	217±114*
	Paretic	80±40	101±49*	141±76*	137±70*	163±85*
Peak Shear Stress (dyne/cm^2^)	Control	27.7±7.3	30.7±8.7	33.0±10.5	37.3±9.8	38.4±9.1
	Non Paretic—Equal Effort	31.1±14.3	32.4±15.1	32.7±12.8	33.9±16.7	37.1±13.1
	Non Paretic—Equal Torque	34.4±15.1	34.6±15.2	36.3±17.5	39.8±23.2	36.5±14.4
	Paretic	36.0±14.5	38.0±15.3	42.6±16.9	41.3±14.4	43.3±15.5

All values are expressed as mean ± SD. NA, Not applicable. The same resting values were used in the equal torque and equal effort conditions for the non-paretic lower limb. Control n = 9; Non Paretic–Equal Effort n = 10; Non Paretic–Equal Torque n = 9; Paretic n = 10. n = number of subjects.

*Significant difference (*p*<0.05) vs. Control–one way ANOVA.

### Cardiovascular Response to Submaximal Contractions of the Knee Extensor Muscles

Blood flow increased in a linear manner with the amount of effort performed in all three limbs tested as shown in Table 3 and S2 Table. There were no differences in heart rate or peak blood flow velocity between groups following submaximal contractions of the knee extensor muscles. For all groups, femoral artery diameter did not change during the protocol; however, the diameter of the artery was significantly greater in control subjects compared to either the paretic or non-paretic limbs of stroke subjects during all conditions. Blood flow (ml/min) through the femoral artery was not different among the groups at rest; however, flow was higher in the femoral artery of control subjects compared to both the paretic and non-paretic limbs (equal torque session) of stroke subjects during all load conditions.

### Change in Femoral Artery Blood Flow in Response to Submaximal Contractions of the Knee Extensor Muscles

**Fig 1:**
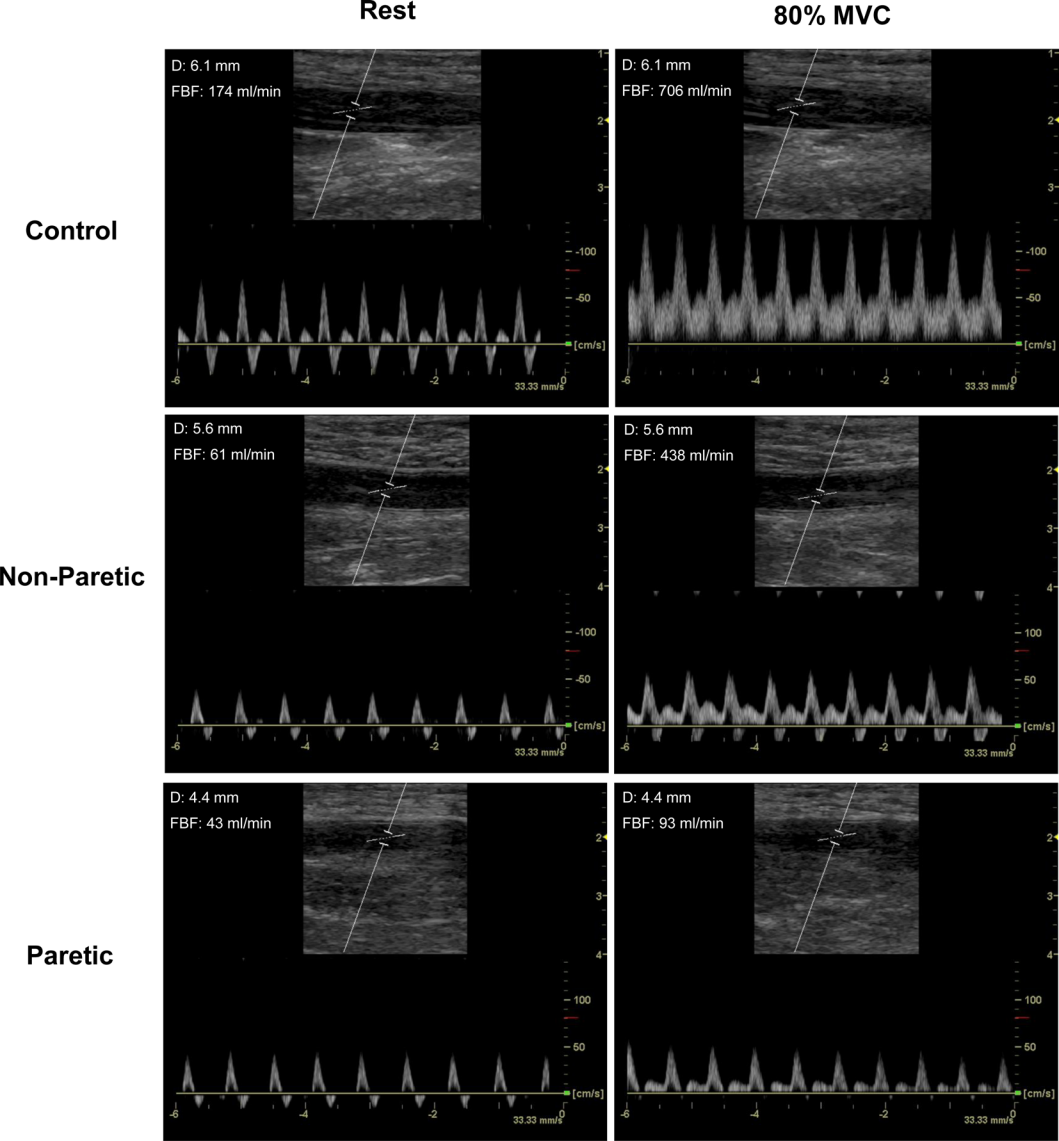
Representative ultrasound images showing blood flow through either the superficial femoral artery of a neurologically intact control subject or the paretic and non-paretic lower limb of a stroke subject at rest or immediately following an 80% MVC. Paretic and non-paretic superficial femoral artery images are from the same subject. D, diameter; FBF, femoral blood flow.

**Fig 2:**
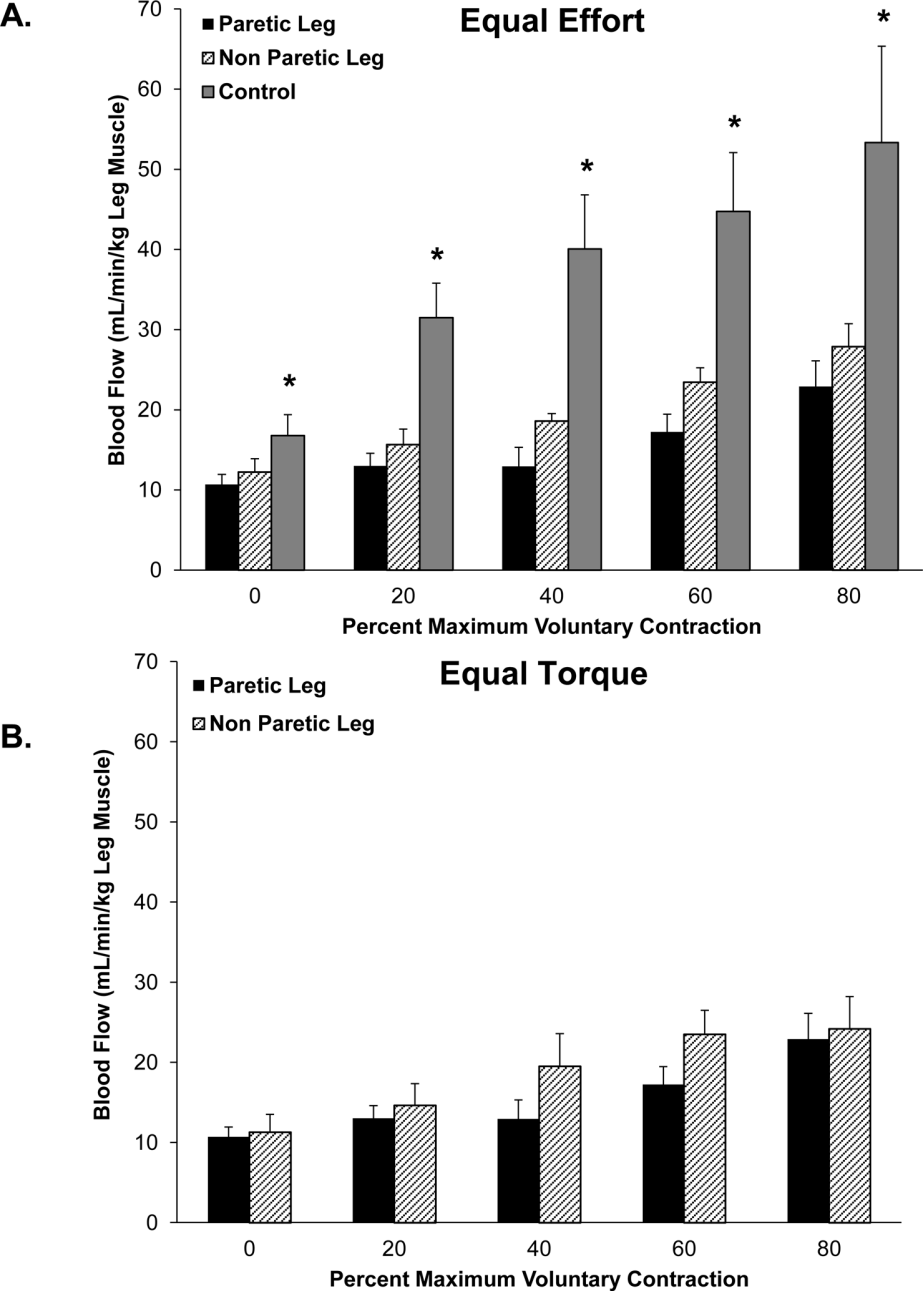
(A) Blood flow through the superficial femoral artery was significantly reduced in the paretic (n = 10) and non-paretic lower limb (n = 10) of stroke subjects in response to 10-second submaximal isometric contractions of the knee extensor muscles compared to age and sex matched control subjects (n = 9). All subjects performed work based on the perceived maximal effort of the test limb (i.e., equal effort). *Significant difference (*p*<0.05) control vs. paretic and non-paretic lower limb, mixed model repeated measures ANOVA. (B) Blood flow through the superficial femoral artery was similar between the paretic (n = 10) and non-paretic (n = 9) lower limb of stroke subjects when the non-paretic limb achieved target torques equal to the paretic limb (i.e., equal torque). Blood flow data could not be quantified in the non-paretic limb of one subject following the equal torque test session. n, number of subjects.

Representative ultrasound images showing blood flow through the superficial femoral artery of a control subject and the paretic and non-paretic lower limb of a stroke subject at rest and immediately following an 80% MVC contraction are shown in Fig 1. As shown in Fig 2A, both the paretic and non-paretic limb of stroke subjects had a reduced hyperemic response to graded muscle contractions compared to control subjects when contractions were performed at equal effort. The paretic and non-paretic limbs of stroke subjects had an equal hyperemic response to graded, submaximal muscle contractions performed during the equal torque test condition (i.e. both limbs generated equal force during each test condition, regardless of differences in limb strength; Fig 2B). The increased hyperemic response to knee extensor muscle contractions in control subjects vs. stroke subjects also was not solely determined by the absolute magnitude of torque generated, as the slope of the response of control subjects was higher than the stroke subjects (*p* = 0.02, S1 Fig).

### The Hyperemic Response to Sub-maximal Contractions of the Paretic Leg Correlates with Leg Function

**Fig 3:**
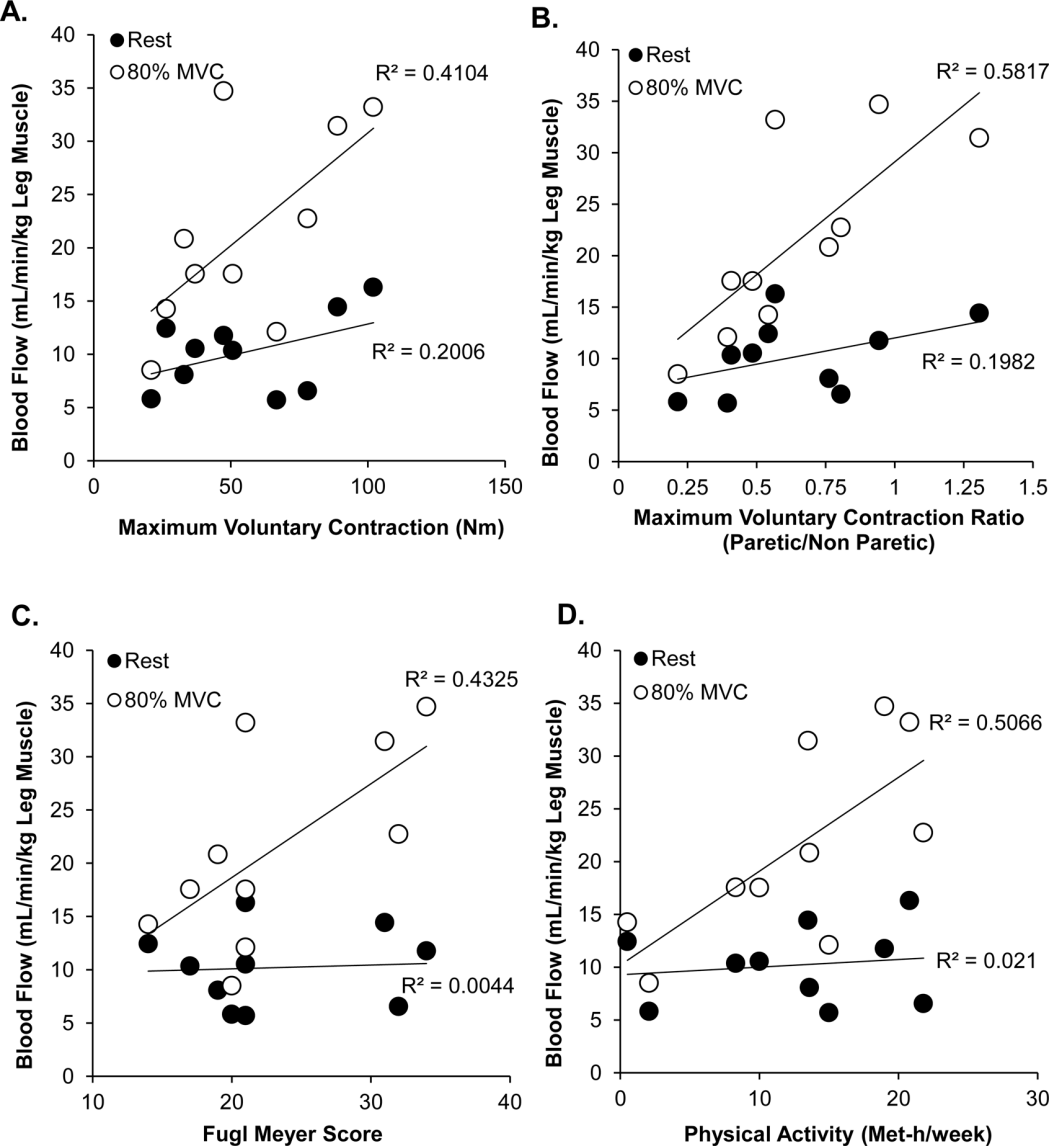
An increased blood flow response in the paretic lower limb following an 80% MVC was positively correlated with (A) paretic limb strength, (B) symmetry of limb strength, (C) Fugl Meyer score and (D) physical activity. There were no correlations between any of the measured parameters and paretic lower limb blood flow at rest.

Increased blood flow through the femoral artery of the paretic limb in response to an 80% MVC positively correlates with 1) paretic lower limb strength (as assessed by maximum torque generated; Fig 3A), 2) the symmetry of strength between the paretic and non-paretic limb (as assessed by the paretic to non-paretic MVC ratio; Fig 3B), 3) the Fugl-Meyer score (a measurement of motor recovery; Fig 3C), and 4) self-reported physical activity (Fig 3D). Resting blood flow in the paretic limb was not correlated with any of the measured parameters, nor was resting blood flow in the non-paretic lower limb (data not shown, *p* > 0.05).

Blood flow through the femoral artery of the paretic lower limb following an 80% MVC was the single best predictor of physical activity (r^2^ = 0.51, β = 0.71, *p* = 0.02), knee extensor MVC ratio between the paretic and non-paretic lower limbs (r^2^ = 0.58, β = 0.76, *p* = 0.01), and Fugl-Meyer score (r^2^ = 0.36, β = 0.658, *p* = 0.039). The single best predictor of the MVC of the paretic limb knee extensor muscles was the blood flow response following the 20% MVC (r^2^ = 0.71, β = 0.845, *p* = 0.002).

## Discussion

This is the first study to assess stroke-related changes in peripheral blood flow regulation in response to submaximal muscle contractions in the chronic stroke population. There are two major novel findings in this study. First, blood flow through the superficial femoral artery in response to equal effort contractions of the knee extensor muscles is significantly reduced in both the paretic and non-paretic lower limb of stroke subjects compared to neurologically intact control subjects. Because blood flow was similar through the superficial femoral artery in the paretic and non-paretic limbs of stroke subjects in response to equal torque knee extensor muscle contractions, this suggests a systemic change in the regulation of peripheral blood flow, rather than a change to the paretic lower limb only. Second, the hyperemic response to muscle contraction in the paretic limb, as opposed to resting measurements, is positively correlated with metrics of limb strength, the symmetry of limb strength between the paretic and non-paretic limbs, Fugl-Meyer score, and levels of physical activity. Though this study included a relatively small number of stroke subjects (n = 10), these findings suggest a potentially important relationship between the peripheral regulation of blood flow in response to exercise/muscle activity and motor function post stroke which warrants further investigation.

Although others have shown that resting blood flow is lower in the paretic lower limb of stroke subjects [1–3] and that resting blood flow can increase in response to therapy,[1] we are the first to quantify the flow response to graded levels of muscle activity. This is important since it provides information in regard to the hemodynamic response to exercise that may be useful in predicting functional outcomes. Data from this study indicate that individuals with stroke demonstrate the ability to increase blood flow to the lower limb in a load-dependent manner. However, the overall magnitude of the response in both limbs is blunted in comparison with controls when contractions are performed at an equal level of perceived effort.

Control subjects did not perform submaximal contractions to absolute torque levels matching those measured in the paretic lower limb of the stroke subject with whom they were age- and sex-matched. Therefore; it is possible that the larger hyperemic response observed in control subjects is a function of them performing more work than those with stroke as the average MVC in control subjects was 54% greater than the non-paretic limb, and 166% larger than the paretic limb of stroke subjects (Table 2). However, as shown in S1 Fig, even when the knee extensor muscles of the control subjects were generating low torque that was similar to values reported in the paretic leg of stroke subjects, hyperemic blood flow was still greater, indicating that the magnitude of hyperemic blood flow is not solely dependent on the amount of work being performed. It is also not likely that the primary mechanism of the blunted hyperemic response observed in stroke subjects is due to atrophy of the muscle because (1) the blunted response occurs in both the paretic and non-paretic lower limbs, (2) the non-paretic lower limb of stroke subjects and the lower limb of control subjects had equal amounts of lean muscle mass, and (3) blood flow was normalized to lean muscle mass of the limb in all groups.

Alternatively, a general lack of physical activity and disuse of the lower limbs could explain the blunted hemodynamic response in both limbs of the stroke subjects. The results of our study indicate that stroke subjects who were the most physically active had the most robust hyperemic response to exercise (Fig 3D). Subjects with stroke often have limited mobility; consequently the amount of daily physical activity they perform can be pathologically low.[12, 13] However, in our study the individuals with stroke and control subjects self-reported similar levels of physical activity (Table 1). Due to the strong positive correlation between physical activity and the hyperemic response to blood flow in the paretic leg, this is an area which warrants more detailed investigation with quantifiable measures of lower body physical activity (for example, using pedometers and accelerometers in an at home setting).

It is also possible that autonomic dysregulation contributes to the blunted hyperemic response in the stroke subjects. It has been shown that after stroke, individuals can have decreased parasympathetic activity with a concordant increase in sympathetic activity,[14–16] which may result in peripheral vasoconstriction. While these types of measurements are beyond the scope of this study, it is worth noting that there were no differences in resting systolic blood pressure and heart rate between stroke subjects and control subjects. Future studies will monitor blood pressure and heart rate during muscle contractions to determine if there is an increased pressor response in stroke subjects.

To our knowledge, a comprehensive study to concordantly examine the effects of altered paretic lower limb blood flow on strength and muscle performance has not been performed, and is necessary to examine a cause-and-effect relationship between changes in paretic lower limb blood flow and limb function. Importantly, data from this study demonstrate a positive relationship between lower limb function and the hyperemic response to muscle contractions in the paretic lower limb, while no relationship exists between lower limb function and resting blood flow values. This highlights important activity-dependent differences in the hyperemic response post stroke that are often overlooked or not characterized. In addition to providing a more telling snapshot of cardiovascular function and physical fitness, this data allows us to speculate that improving the hyperemic response to exercise could help optimize motor recovery. Current stroke rehabilitation strategies primarily focus on lower limb strength, movement quality, and cardiovascular fitness as separate issues. Data from this study suggest that examining the blood flow response to a single muscle contraction may potentially be used as another assessment tool of impairment/cardiovascular health during the rehabilitation process. Future studies will examine the hyperemic response to longer contractions that may more closely mimic strengthening regimens or activities of daily living.

### Study Limitations

Subjects were not in a fasting state during this study. Studies which assess endothelial function of conduit arteries using the flow mediated dilation (FMD) technique recommend a minimum 8 hour fast prior to performing the technique as the postprandial phase can reduce peripheral endothelial function.[17] Though we did not specifically test endothelial function with FMD, it is conceivable that a non-fasting state could contribute to variation within our measurements of active hyperemia. However, the stroke subjects served as their own controls and were tested at the same time of the day, thus day-to-day variation should be minimal assuming no change in diet.

Subjects also did not abstain from medications. While it would be advisable for subjects to discontinue medication prior to assessment of endothelial function, we chose not to require the participants to discontinue medications they used for the medical management of stroke. A list of medications the stroke subjects were taking is listed in S1 Table (note: control subjects were not taking any medication).

The distance from the bifurcation of the femoral artery into the deep and superficial branches was also not measured, however based on the location of the artery in the thigh, the artery depth, and the observed diameter being consistent with published values,[18] we concluded that the superficial femoral artery was visualized.

The results of this study indicate that the hyperemic response to graded muscle contractions is reduced in both the paretic and non-paretic limb of stroke subjects compared to control subjects; however, it cannot be concluded whether the reduced blood flow is either contributing to the muscle weakness or is a consequence of the weakness of the knee extensor muscles. S1 Fig indicates that although the hyperemic response in the paretic limb is still linear, the slope of the response is less than controls (*p* = 0.02). Still, future larger studies which modulate blood flow to the limb are necessary to determine if a cause-and-effect relationship exists between limb blood flow and knee extensor muscle force generating capabilities.

Finally, this study has a relatively small sample size. Future studies with a larger sample size will allow better examination of the relationship between blood flow and function by controlling for co-variance among independent measures.

## Supporting Information

S1 Table Medications taken by all stroke subjects. Control subjects were not taking any medications.
(DOCX)

S2 Table Maximum voluntary contraction, lower limb muscle mass, and femoral artery blood flow values at each contraction level of all subjects. Scatter plots indicate that the hyperemic response to muscle contractions increased linearly with increased torque in all groups (r^2^ ≥ 0.92).
(XLSX)

S1 Fig Blood flow increased linearly with torque generated by the knee extensor muscles in control subjects (closed circles) and in the paretic limb of stroke subjects (open circles). While control subjects generated higher torque levels than stroke subjects, blood flow was still greater in control subjects at torque levels comparable to those measured in stroke subjects.
(TIF)
