# Supplementary material for: Impaired Hyperemic Response to Exercise Post Stroke
Source: PLoS One. 2015 Dec 2;10(12):e0144023. doi: 10.1371/journal.pone.0144023 (PMC4667998; doi:10.1371/journal.pone.0144023)
Supplement: S1 Table — Control subjects were not taking any medications. (DOCX) [file pone.0144023.s001.docx]

| **Table S1. Medications taken by participants.** | |
| --- | --- |
| **Subject** | **Medication Taken** |
| S203 | Clopidogrel, Aspirin, Divalproex, Ezetimibe, Sertraline, Zolpidem, Primidone, Tamsulosin |
| S204 | Clopidogrel, Temazepam, Fluticasone, Rosuvastatin |
| S205 | Olmsartan, Aspirin |
| S206 | Metformin, Rosuvastatin, Lisinopril, Cyclobenzaprine, Alprazolam |
| S207 | Amlodipine |
| S208 | Aspirin, Simvastatin, Atenolol, Hydrochlorothiazide |
| S209 | Sertraline |
| S210 | None |
| S212 | None |
| S213 | Baclofen |
