# Supplementary figures and images for: Impaired Hyperemic Response to Exercise Post Stroke
Source: PLoS One. 2015 Dec 2;10(12):e0144023. doi: 10.1371/journal.pone.0144023 (PMC4667998; doi:10.1371/journal.pone.0144023)

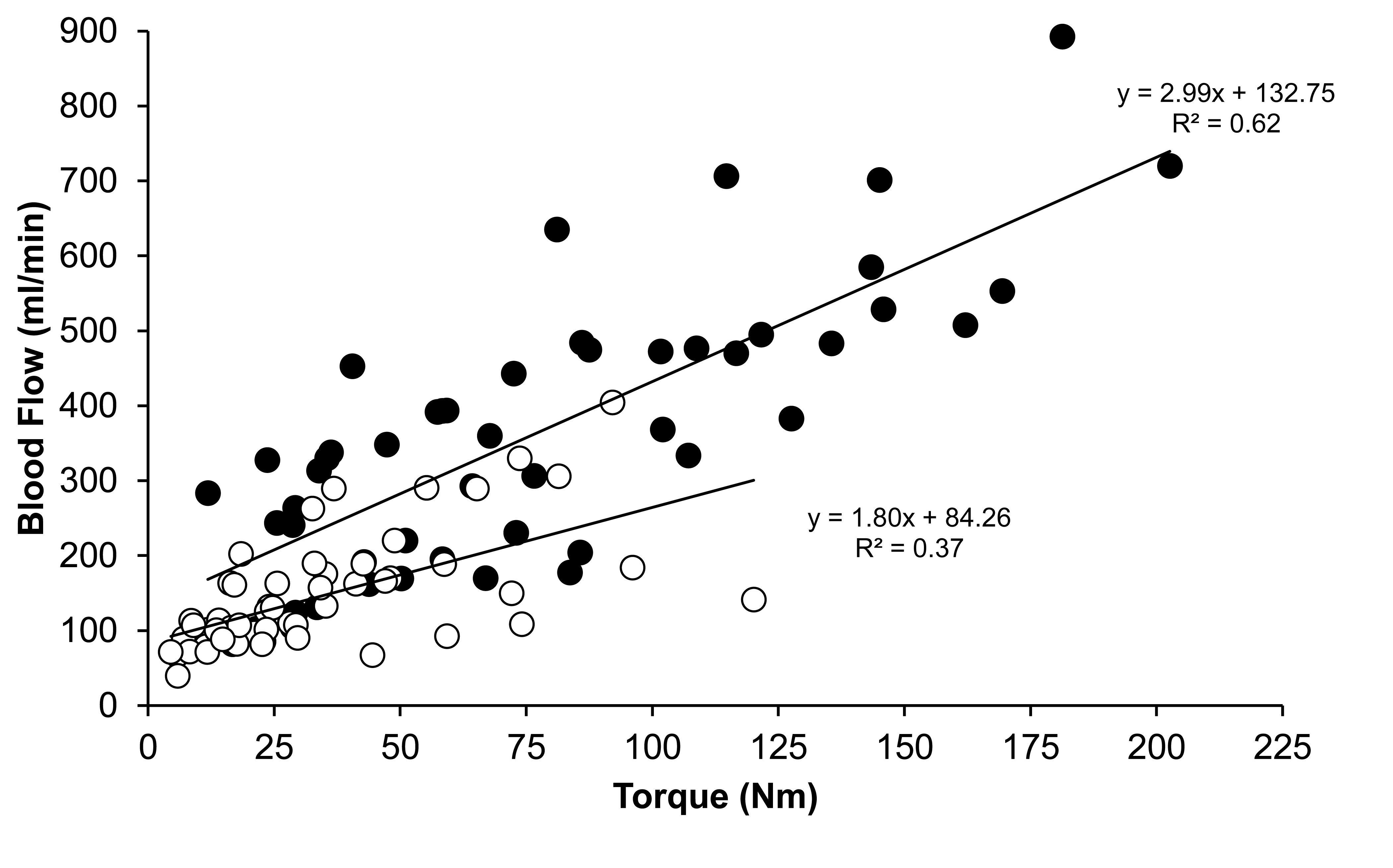

Supplement: S1 Fig — While control subjects generated higher torque levels than stroke subjects, blood flow was still greater in control subjects at torque levels comparable to those measured in stroke subjects. (TIF) [file pone.0144023.s003.tif]
